# Epileptic seizure biophysics: the role of local voltage difference

**DOI:** 10.1186/s40779-025-00620-4

**Published:** 2025-07-11

**Authors:** Kui-Ying Yin, Tao Yu, Chuan Liu, Jin-Rong Yin

**Affiliations:** 1 Link Sense Laboratory, Nanjing Research Institute of Electronic Technology, Nanjing, 210039 China; 2 Beijing Institute of Functional Neurosurgery, Xuanwu Hospital of Capital Medical University, Beijing, 100053 China

**Keywords:** Epilepsy, Epileptic seizures, Slow-varying direct current (Sv DC) field, Local voltage difference, Epileptogenic zone

## Abstract

**Background:**

Epilepsy is a neurological disorder characterized by recurrent seizures due to hyperexcitable neuronal network activity. The manifestations vary widely, ranging from subtle sensory disturbances to profound alterations of consciousness, depending on which brain regions are affected and their underlying etiology. Exploring the biophysical mechanisms of epileptic seizures holds significant for predicting and controlling the disease.

**Methods:**

We analyzed 45 spontaneous seizures recorded from 24 patients with focal epilepsy, as well as stimulation-induced seizures from 2 additional patients. A second-order Butterworth low-pass filter isolated the slow-varying direct current (Sv DC) component (0.01–0.5 Hz), a frequency range often overlooked in electroencephalography. The energy ratio of the Sv DC component was calculated by dividing its total energy by the total signal energy during seizures and over a 1-hour period including the seizure, enabling comparison between ictal and interictal states.

**Results:**

The Sv DC component exhibited spatially dynamic changes during both ictal and interictal periods and showed a moderate correlation with high-frequency activity. Moreover, it accounted for a high energy proportion in both periods, with seizure data showing that 80.82% of leads had ≥ 60% Sv DC energy. Notably, interictal Sv DC fluctuations were more pronounced in electrodes located within the epileptogenic zone, suggesting its potential as a marker for epileptogenic localization. Furthermore, the temporal variability of the Sv DC signal, reflected in its dispersion, demonstrates potential as an early indicator of seizure development.

**Conclusions:**

The Sv DC component may reflect local voltage differences likely linked to ion channel activity, potentially contributing to seizure initiation. Combined analysis of Sv DC with low- and high-frequency components offers a comprehensive framework for understanding epileptic networks and guiding diagnosis and therapy.

**Supplementary Information:**

The online version contains supplementary material available at 10.1186/s40779-025-00620-4.

## Background

Epilepsy is a common chronic neurological disorder with a documented history spanning over 2 millennia [1], characterized by persistent seizure tendencies that adversely affect neurobiological functions, cognitive abilities, psychological well-being, and social interactions [2]. Epilepsy incidence peaks twice across the lifespan: during infancy (< 1 year) and late adulthood (> 50 years) [2]. The overall lifetime prevalence is 7.60 per 1000 individuals [3]. In older adults, epilepsy is often associated with stroke and neurodegenerative diseases, while in younger populations, it is one of the most common neurological disorders [4].

The aetiology of epilepsy is highly complex: structural abnormalities in the brain, genetic metabolic disorders, infections, injuries, tumors, and ischaemia are all precipitants of seizures. Although the mechanisms underlying epilepsy exhibit considerable diversity, the core pathological process involves the transition of neural circuits into a hyperexcitable state that promotes spontaneous and recurrent seizure activity. This transformative process is often linked to an imbalance between excitatory and inhibitory activities within neuronal networks [5]. In addition, multiple factors may also contribute to epileptogenesis, including gliosis, neuroinflammation, and genetic mutations, among others. Consequently, epilepsy should not be simply defined as a singular disease entity, but rather recognized as a manifestation of pathological neural hypersynchrony resulting from diverse etiological factors. Therefore, investigating the specific mechanisms underlying this abnormal electrical activity may hold significant potential for neurostimulation for the treatment of epileptic seizures.

Some studies have attempted to elucidate the mechanisms of epilepsy formation from multiple dimensions, such as neurostructural morphology, genetics, and molecular mechanisms, through neuroimaging [6], genetics [7], and pathology [8], with the aim of exploring treatments that target its individualized etiology. However, the exact mechanism through which the brain generates abnormal electrical activity of sufficient magnitude for triggering seizures remains a major challenge. Therefore, biophysical models may provide a means of identifying common seizure features across different causes, which could facilitate the exploration of universal therapeutic approaches. Although biophysical models have been used to explore the roles of specific neurophysiological processes responsible for seizure transitions, such as intracellular and extracellular K^+^ dynamics, transmembrane chloride gradients, and calcium-activated processes [9–11], it is still difficult to explain the evolution of ictal activities through any single biophysical mechanism [12].

Electroencephalography (EEG) is a crucial tool for elucidating the discharge characteristics of epileptic seizures in the brain [13]. Since its development in the early twentieth century, EEG has played a crucial role in diagnosing epilepsy and localizing seizure origins. In recent years, intracranial electrode recordings, especially stereo-electroencephalography (SEEG) recordings, have provided brain electrical activity data with higher spatiotemporal resolution for the diagnosis and treatment of epilepsy. These high signal-to-noise ratio signals provide favourable conditions for the study of high-frequency activities, causing high-frequency oscillations (HFOs) to become a focal point in epilepsy research. Numerous studies in recent years have confirmed that interictal HFOs can be considered important markers of the epileptogenic zone [14, 15], and low-voltage fast activities (LVFAs) during seizures are crucial features of seizure onset [14, 16]. However, the complexity of epileptic activity is not limited to high-frequency signals, as low-frequency components, including low-frequency direct current (DC) components, also play a significant role in epileptic seizures but have received relatively little attention. Existing research [16] has described this phenomenon and proposed the concept of ictal direct current shifts (icDCs), confirming their substantial value in locating epileptogenic zones. [17, 18]. Due to their low specificity and susceptibility to interference, single-channel DC activities, unlike HFOs, have not become established markers for epileptogenic zones. However, since single-channel DC activities also change during seizures, we are interested in whether systematic changes can be observed when multiple surrounding channels are analysed jointly. To address this,the study aimed to investigate whether joint analysis of the DC component with low-frequency brain rhythms (1–4 Hz) and high-frequency activities (40–300 Hz) across multiple channels could reveal consistent spatial and temporal patterns related to seizure dynamics.

## Methods

### Data sources

We reviewed data acquired from patients with drug-resistant epilepsy who underwent SEEG during presurgical evaluation at the Beijing Institute of Functional Neurosurgery between February 2022 and June 2024. This study was conducted according to the guidelines of the Declaration of Helsinki and has been approved by the Medical Ethics Committee of Xuanwu Hospital, Capital Medical University (LYS [2022] 012). All patients provided informed consent after admission. This retrospective analysis used existing data from patients who had completed treatment, with no additional data collection. All data retrieval methods were conducted in accordance with the enrolment and exclusion criteria established in this study. Patients with focal epilepsy were eligible for enrolment if their presurgical evaluation included: 1) high-quality continuous intracranial electroencephalography (iEEG) recordings obtained using a video-EEG monitoring system (Neuracle, China) with a sampling rate of 1024 Hz, lasting for at least 5 d and encompassing sleep periods; 2) documentation of at least 2 habitual seizures; and 3) availability of presurgical brain high-resolution magnetic resonance imaging (MRI), ^18^F-fluorodeoxyglucose positron emission tomography, and post-electrode implantation brain computed tomography (CT) scans. The study size was determined by the number of eligible patients available during the study period. Exclusion criteria primarily included patients with a history of intracranial surgery or those with suboptimal study quality. Suboptimal study quality was defined by low sampling rates in iEEG recordings, data interruptions, highly disorganized background activity, frequent epochs of activity on the interictal-ictal continuum, and low-resolution results in pre-surgical or postsurgical imaging studies. More details are presented in Additional file 1: Clinical data collection.

### EEG signal processing

#### EEG signal preprocessing and data selection

The raw EEG data was preprocessed using digital filters: a 50 Hz notch filter was applied to eliminate line noise interference, followed by a 0.01−300 Hz bandpass Butterworth filter (4th-order) to retain signals within the effective frequency range. Subsequently, all channels were visually inspected for artifact identification and screening. Channels exhibiting significant baseline drift (continuous amplitude shifts exceeding ± 100 μV) or noise caused by the poor electrode contact (e.g., sudden high-amplitude fluctuations or signal discontinuities) were flagged as invalid and excluded. The remaining channels with no observable artifacts (e.g., electromyographic interferences, eye movements, or motion artifacts) and stable signals were retained as valid data for analysis. All preprocessing procedures were implemented using the EEGLAB toolbox (version 2021.1), with cross-validation performed independently by 2 researchers to ensure consistency.

#### DC component filter design

To separate the 0.01−0.5 Hz DC component from the brain electrical signals, we employed a Second-Order Butterworth lowpass filter. The cut-off frequency of the filter was set to 0.5 Hz. Although the filter exhibits a gradual roll-off rather than an abrupt cut-off, signals beyond this frequency are significantly attenuated, retaining only minimal residual energy that does not influence the overall trend of the results. After the original brain electrical signals were input into this filter, the DC component within the desired frequency band was retained, while high-frequency noise > 0.5 Hz was filtered out. This design helped to extract the low-frequency DC component in brain electrical signals for subsequent analysis.

#### Calculation of the DC energy ratio

To assess the energy ratio of the DC component during epileptic seizures, we selected data segments from 45 epileptic seizures, each segment spanning 2 min before and 2 min after the onset of the seizure. First, we calculated the squared values of the DC component in each data segment and summed them to obtain the total energy of the DC component. Then, we calculated the sum of the squares of the entire signal segment, which represented the total energy of the signal segment. Finally, we divided the total energy of the DC component by the total energy of the signal segment to obtain the energy ratio of the DC component during epileptic seizures. Similarly, we calculated the energy ratio of the slow-varying direct current (Sv DC) component for 1 h of data, including the 4 min seizure period. This calculation process allows us to understand the differences in the Sv DC component ratio between the seizure period and the interictal period, helping to reveal how the Sv DC plays a dominant role in epileptic seizures.

### Related calculations

#### Autocorrelation function

The autocorrelation function is a measure of the degree of correlation between a signal and a time-delayed version of the same signal. The autocorrelation function $\rho \left(\tau \right)$ or a signal $s\left(t\right)$ is defined as formula (1):

1 $$\rho \left( \tau \right) = \frac{{\smallint s\left( t \right) \cdot s\left( {t + \tau } \right)dt}}{{\smallint s^{2} \left( t \right)dt}}$$

where $t$ is the time variable, and $\tau$ is the time-shift variable.

#### Cross-correlation coefficient

The cross-correlation coefficient quantifies the degree of correlation between 2 different signals. The cross-correlation coefficient $\rho_{12}$ between 2 signals ${s}_{1}\left(t\right)$ and ${s}_{2}\left(t\right)$ is defined as formula (2):

2 $$\rho_{12} = \frac{{\smallint \left[ {s_{1} \left( t \right) - \overline{s}_{1} } \right] \cdot \left[ {s_{2} \left( t \right) - \overline{s}_{2} } \right]dt}}{{\sqrt {\smallint \left[ {s_{1} \left( t \right) - \overline{s}_{1} } \right]^{2} dt} \cdot \sqrt {\smallint \left[ {s_{2} \left( t \right) - \overline{s}_{2} } \right]^{2} dt} }}$$

where $t$ is the time variable, and $\overline{s}_{1}$ and $\overline{s}_{2}$ are the average values of the signals ${s}_{1}\left(t\right)$ and ${s}_{2}\left(t\right)$, respectively.

#### Brain network construction

To assess the neuronal synchrony between different cortical regions of the brain, we adopted a phase synchronization method of brain electrical signals. This method can detect weak phase relationships between signals without being affected by instantaneous amplitude changes. The phase locking value (PLV) is an effective tool for analysing brain phase synchronization. PLV is particularly suitable for studying neural synchrony as it captures both normal fluctuations in brain activity and pathological changes, such as the highly synchronized discharges characteristic of epileptic seizures [19]. Its sensitivity to these dynamics makes it an effective tool for distinguishing between normal and epileptic states, providing valuable insights into brain network functionality [20]. The PLV is defined as the average of the absolute values of the phase difference between 2 signals, and its calculation as formula (3):

3 $${\text{PLV}}_{t} = \frac{1}{N}\left| {\mathop \sum \limits_{n = 1}^{N} exp\left( {j\theta \left( {t,n} \right)} \right)} \right|$$

where $\theta (t,n)$ is the phase difference between the 2 signals, $N$ is the signal length, and $\mathit{exp}(j\theta (t,n))$ is the complex signal obtained via Euler’s formula. The PLV ranges from 0 to 1. When the phases of the 2 signals are completely synchronized, the PLV is 1; when the phases are completely out of sync, the PLV is 0. By calculating the PLV between signals from different brain regions, we can construct a brain network, revealing the patterns and dynamic changes in brain functional connectivity.

### Statistical analysis

Descriptive statistics were first computed for all variables, including the mean ± standard deviation (for normally distributed data) or the median and interquartile range (for non-normally distributed data). Normality of the data was assessed using the Shapiro-Wilk test in SPSS version 21. For data that did not meet the assumption of normality, non-parametric comparisons were conducted using the Wilcoxon rank-sum test in GraphPad Prism version 9.0. A *P*-value < 0.05 was considered statistically significant.

## Results

### Demographic and clinical characteristics of the patient cohort

The study population consisted of 24 patients (15 males and 9 females) with recorded spontaneous seizures [age: (27.2 ± 9.3) years; epilepsy duration: (13.3 ± 9.0) years], as well as 2 additional patients (J and H) who experienced stimulation-induced seizures. Among the 24 patients with spontaneous seizures, anonymized labels such as “patient W” and “patient B” were used for illustrative purposes in the figures and analysis. Of these patients, 18 exhibited secondary generalized tonic-clonic seizures, while 6 had focal seizures. MRI revealed lesions in 9 patients, with locations including the frontal lobe, temporal lobe, parietal lobe, occipital lobe, and hippocampus. The remaining 15 patients showed no detectable lesions on MRI. Intracranial EEG recordings were performed using a varying number of electrodes and contacts, with the seizure onset zone identified in different brain regions, including the frontal lobe, temporal lobe, parietal lobe, occipital lobe, insular lobe, central region, hippocampus, and anterior cingulate gyrus. Surgical interventions included radiofrequency ablation and resection, with pathological findings indicating focal cortical dysplasia in 8 patients, mild malformations of cortical development in 5 patients, gliosis in 3 patients, and ganglioglioma in 1 patient. The demographic and clinical data for the patient cohort are summarized in Table 1. This cohort provides a comprehensive representation of patients with diverse epilepsy profiles, enabling a detailed analysis of Sv DC shifts and their potential role in seizure onset. Two additional patients (patients J and H) were included to demonstrate seizures induced by electric cortical stimulation, with their demographic and clinical data presented in the last 2 rows of Table 1.

**Table 1:** Demographic and clinical data for the patient cohort

Patient No	Gender	Age (years)	Epilepsy duration (years)	MRI lesion location	Seizure type	Electrodes/contacts	Lead in SOZ	Surgery	Pathology
1	M	10	5	B PL	SGTCS	10/114	R or L PL	RF: B PL	–
2	M	43	29	N	SGTCS	14/204	R OL	RF & RS: R OL	FCD IIa
3	M	17	9	N	SGTCS	5/76	R OL	RS: R OL, Hippo	FCD Ia
4	F	22	3	N	Focal	10/118	L CR	RF: L CR, MCG, PCG	–
5	F	41	37	N	SGTCS	8/116	R IL	RS: R IL	FCD IIb
6	M	16	4	L FL	Focal	10/114	L IL	RF & RS: L CR, FL, IL	Gliosis
7	M	26	17	N	Focal	9/126	L TL	RF: L TL, PL	–
8	M	26	23	N	SGTCS	9/132	L TL & PL	RS: L TL, PL	FCD IIa
9	M	28	9	N	SGTCS	12/180	L FL	RS: L FL	FCD IIb
10	F	31	4	L PL, OL	Focal	12/184	L PL	RF: L PL, TL	–
11	F	23	13	L TL	SGTCS	9/132	L TL & OL	RS: L TL	FCD Ia
12	M	23	22	L Hippo	SGTCS	11/170	L IL	RS: L IL, FL	FCD IIa
13	M	36	10	N	Focal	15/210	L Hippo	RF: L TL, IL	–
14	M	26	14	N	SGTCS	10/150	R Hippo	RS: R TL, FL	mMCD
15	F	25	11	N	SGTCS	7/100	L TL	RF & RS: L TL, IL, PL	Gliosis
16	F	20	11	N	SGTCS	10/138	L Hippo	RS: L TL, IL	mMCD
17	M	13	1	R TL	Focal	15/242	R IL	RS: R TL, IL	GGs
18	M	37	13	L Hippo	SGTCS	8/124	L TL	RS: L TL	mMCD
19	F	26	9	L TL, OL	SGTCS	14/214	L TL	RF: L TL, IL, OL	–
20	F	32	15	N	SGTCS	8/102	L Hippo	RS: L TL	Gliosis
21	M	21	5	N	SGTCS	9/140	R IL	RS: R IL, TL	mMCD
22	M	48	10	N	SGTCS	11/172	Extensive	No RS	–
23	F	31	21	N	SGTCS	8/114	L ACG	RS: L ACG, FL	mMCD
24	M	31	25	R FL	SGTCS	8/116	R FL	RS: R FL	FCD IIb
J	M	39	11	R PL	SGTCS	18/262	R PL	RF: R PL	–
H	M	20	12	N	SGTCS	12/182	R PL	RF: R PL	–

“–” indicates that no pathological result is available. *M* male, *F* female, *N* negative, *R* right, *L* left, *B* bilateral, *SGTCS* secondary generalized tonic-clonic seizure, *RF* radiofrequency ablation, *RS* resection, *FCD* focal cortical dysplasia, *mMCD* mild malformations of cortical development, *FL* frontal lobe, *TL* temporal lobe, *PL* parietal lobe, *OL* occipital lobe, *IL* insular lobe, *CR* central region, *Hippo* hippocampus, *MCG* midcingulate gyru, *PCG* posterior cingulate gyru, *ACG* anterior cingulate gyru, *SOZ* seizure onset zone, *GGs* ganglioglioma *MRI* magnetic resonance imaging

### Settings and key findings in the EEG

**Fig. 1:**
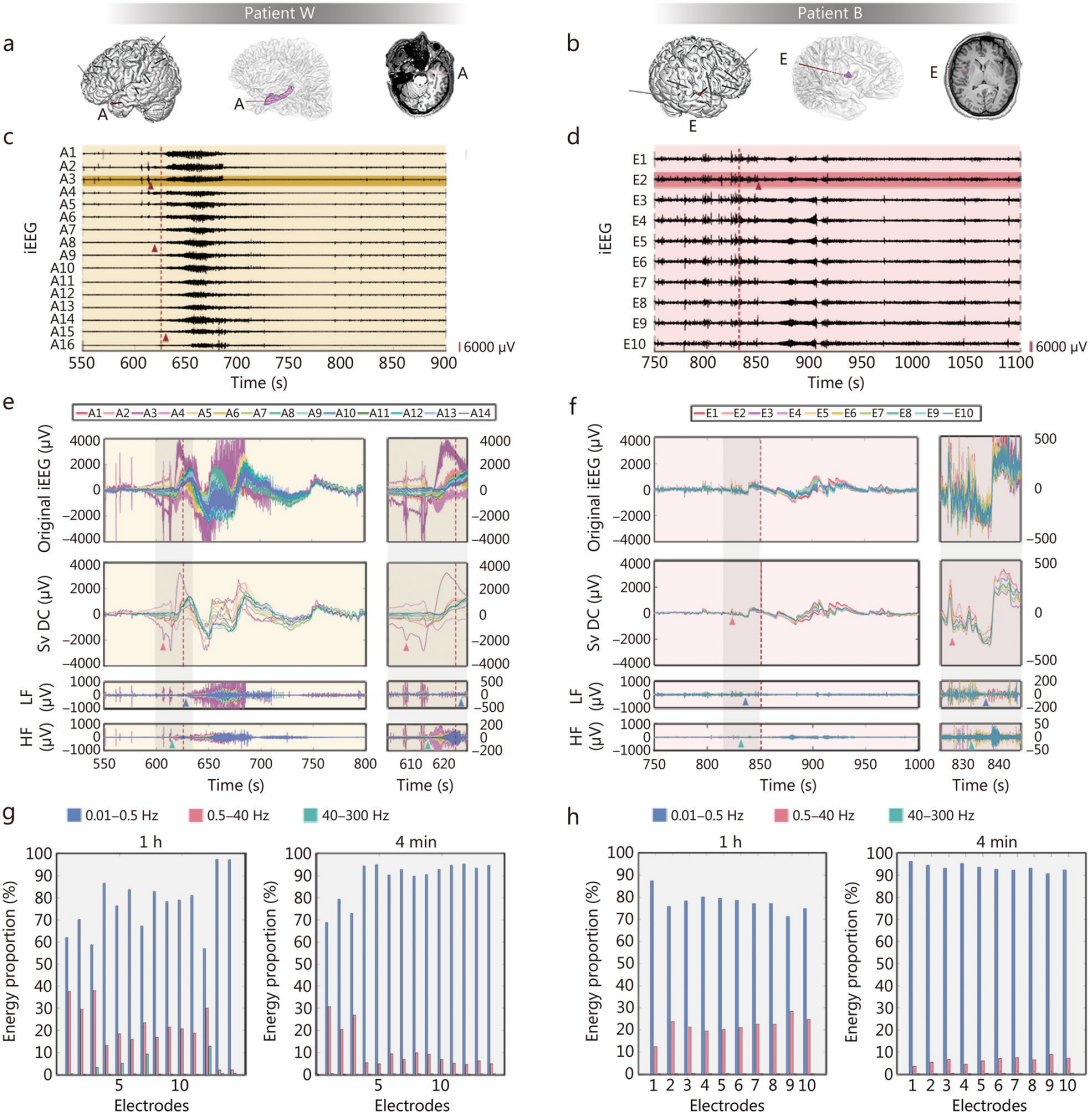
Intracranial EEG signal characteristics of natural seizures. The intracranial electrode locations of patient W (**a**) and patient B (**b**). The implanted SEEG electrodes, the epileptogenic zones (the hippocampus in patient W and the insula in patient B, purple) and the trajectory of the seizure onset electrode were shown, respectively. The lead A3 and E2 are located within the epileptogenic zones, respectively. The ictal intracranial electroencephalography (iEEG, 0.5−300 Hz) on the seizure onset electrode in patients W and B. The low-voltage fast activity (LVFA, red triangles) originating from A3 progressively accumulated to all other leads (**c**), the burst of LVFA originating from E2 (red triangle) rapidly expanded to all leads (**d**). The amplitude scale is shown in the lower right corner. The red dashed line shows the moment of seizure onset of clinical visual analysis. The ictal iEEG on the seizure onset electrode (A/E) in patient W (**e**) and patient B (**f**). Full-band iEEG (original iEEG, 0.01−300 Hz), as well as slow-varying direct current (Sv DC, 0.01−0.5 Hz), low-frequency (LF, 0.5−40 Hz), and high-frequency (HF, 40−300 Hz), are respectively listed. The Sv DC dispersed (red triangles) apart in both patients earlier than the onset of LF (blue triangles) and HF (green triangles) activities. The red dashed line shows the onset. The energy proportion of different frequency bands of EEG on the seizure onset electrodes of patient W (**g**) and patient B (**h**), respectively. The left panels in both figures show the energy proportion of each lead in 1 h EEG data (including the ictal period), and the right panels show the energy proportion of each lead in a 4 min ictal period. Blue for the Sv DC, red for the 0.5−40 Hz, green for the 40−300 Hz. Leads A15 and A16 in Fig. 1e and g were removed due to artifacts. EEG electroencephalography, SEEG stereo-electroencephalography, iEEG intracranial electroencephalography

To systematically analyse brain electrical signals, we adopted a Second-Order Butterworth lowpass Filter with a cut-off frequency of 0.5 Hz, focusing on the 3 frequency bands of 0.01−0.5 Hz, 0.5−40 Hz, and 40−300 Hz, and conducted a thorough investigation of patients’ brain electrical signals during seizures. Figure 1 shows the key findings in the segmented frequency EEG analysis for patient W and patient B. The electrode positions and epileptogenic zones of these 2 patients are shown in Fig. 1a and b. During patient W’s seizure, the red triangle in Fig. 1c marks the initial appearance of LVFA in lead A3, followed closely by low-frequency rhythmic activity, which gradually spread to a larger range of leads. The spatiotemporal pattern of LVFA propagation in this patient aligns with the typical neural pathway of seizure spread. However, during patient B’s seizure (Fig. 1d), the LVFA emerged explosively over a wide range, rapidly expanding to all leads, with almost no observable time delay in LVFA onset between different leads. Importantly, despite the differences in seizure propagation patterns between the 2 patients, a stable and consistent pattern of change in the 0.01−0.5 Hz range can be observed in both patients.

### Proposal of the Sv DC field

On the basis of the aforementioned observations, we found that the 0.01−0.5 Hz DC component exhibited shift and dispersion phenomena during epileptic seizures. This change is not only reflected in the electrical activity on the same electrode but also shows pronounced dispersion among different leads. When the seizure ends, these shifted and dispersed 0.01−0.5 Hz DC components re-converge to the baseline level (Fig. 1e, f). Therefore, the shifted and dispersed 0.01−0.5 Hz DC components are not an isolated point issue but rather a problem of electric field changes. Unlike the DC, this component exhibits slow-varying bioelectric behavior, and these slow variations lead to potential differences at different locations in the brain. Here, we define this issue by characterizing the DC component as a constant component (i.e., the average value within that time period) of the brain electrical signal over a short time interval (the DC is set to 2 s in this paper). The DC component defined in this manner is equivalent to the frequency component within the 0.01−0.5 Hz bandwidth over the entire time period. To distinguish it, we define this brain electrical component as the Sv DC field.

We conducted a detailed analysis of 45 epileptic seizures across 24 patients and observed a stable and consistent effect, namely, the shift and dispersion of the Sv DC. This behavior is clearly demonstrated in Additional file 1: Fig. S1, confirming that it is a stable feature of EEG during epileptic seizures. The following are several stable characteristics of this phenomenon. (1) Temporal sequence: the shift and dispersion of the Sv DC significantly precede the rhythmic changes in EEG features, indicating the onset of epileptic seizures, as confirmed by traditional visual analysis. This observation suggests that voltage differences are a prerequisite for epileptic seizures, meaning that voltage changes occur first, followed by the visible rhythmic activity on the EEG. (2) Lesion localization: in terms of spatial distribution, the dispersion initially appears in the epileptogenic zone, whereas areas farther from the lesion exhibit dispersion only later. This pattern is highly valuable for accurately locating the epileptogenic zone, helping clinicians to identify the lesion location more precisely. (3) Degree of change: on electrodes directly recording epileptogenic activity, the dispersion is more pronounced; in contrast, on electrodes farther from the lesion, the dispersion is weaker. This observation indicates that the Sv DC exhibits more prominent bioelectric changes in the lesion area, further emphasizing the crucial role of the lesion in epileptic seizures. (4) Seizure process: as an epileptic seizure progresses, the degree of Sv DC dispersion gradually peaks. At the end of the seizure, these dispersed Sv DC signals re-converge to the baseline level, and the potential differences gradually return to stable levels after the energy transformation concludes. This dynamic change process is visually demonstrated in Additional file 1: Fig. S1.

### Characteristics of seizure onset

Our observations revealed that the gradual dispersion of the Sv DC component is the earliest electrical activity feature at the onset of epileptic seizures, preceding the outbreak of LVFA, low-frequency rhythmic activity, and the onset point determined by electrophysiologists through the conventional visual inspection of EEG (Fig. 1e, f). Prior to seizure onset, DC fluctuations and high-frequency burst activity often occur. The dynamic process subsequently induces voltage generation in surrounding brain regions, and the resulting induced currents spread within the brain tissue. The differential diffusion of these induced currents in grey matter vs. white matter results in the aforementioned distinct features of seizure discharge propagation (as illustrated in patients W and B in Fig. 1).

### Sv DC dominates epileptic activity

The energy proportions of the following three frequency bands were calculated from the seizure onset electrodes of patients W and B: Sv DC (0.01–0.5 Hz), low-to-mid frequencies (0.5–40 Hz), and high-frequency activity (40–300 Hz). The energy proportion of each lead in 1 h EEG data (including the ictal period), and a 4 min ictal period were compared (Fig. 1g, h). Furthermore, we analysed the EEG data from 45 seizure episodes across 24 patients, with each episode spanning 4 min (2 min before and after seizure onset to sufficiently encompass the seizure, as seizures typically last 1−2 min). The results showed that, among the total 6360 leads from 476 implanted SEEG electrodes, 5140 leads (80.82%) had an Sv DC energy proportion ≥ 60%, and 2589 leads (40.71%) had an energy proportion ≥ 90%. Upon analysing the brain electrical data for 1 h, including these 4 min seizure episodes, we found that 4400 leads (69.18%) had an Sv DC energy proportion ≥ 60%, and 1304 leads (20.50%) had an energy proportion ≥ 90% (Additional file 1: Fig. S2; Additional file 2: Tables S1, S2).

From a systems theory perspective, this finding suggests that the Sv DC component plays a prominent role in the overall system, potentially exerting a substantial influence on the system’s properties and functions. Given its high energy proportion, the Sv DC component is an important factor in determining the system’s characteristics, affecting its overall performance, stability, response speed, and other properties. Comparison between the 1 h period encompassing the seizure and the 4 min seizure period also revealed a notable shift in energy state dynamics during the seizure (Additional file 1: Fig. S2). High-energy proportion components significantly influence the states and behaviors of other components through interactions and energy transfers, thereby exerting profound effects on the entire system’s operation. Therefore, to analyse and optimize the system, it is essential to fully consider the role of this high-energy proportion component.

### Sv DC changes in the interictal period

**Fig. 2:**
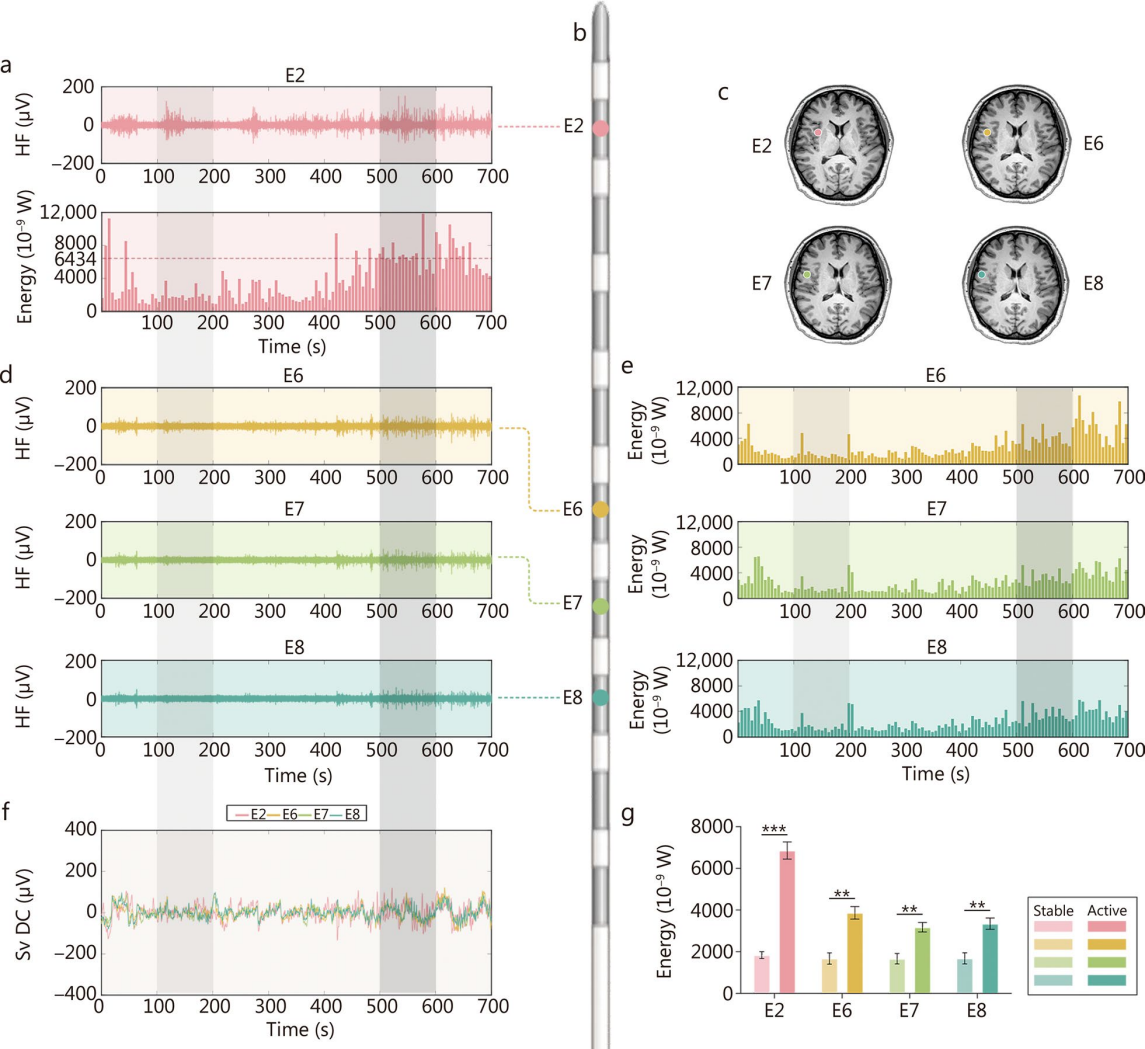
The energy changes in the interictal intracranial electroencephalography (iEEG, taking patient B as an example). **a** The interictal high-frequency (HF) iEEG recorded in the E2 lead (in the insula epileptogenic cortex). The panel below shows the full-band energy changes in the E2 lead during the same period. The red dashed line represents the mean energy value. The energy in the light gray region (100−200 s) is relatively low, and that in the dark gray region (500−600 s) is higher. **b** Schematic diagram of the E electrode (containing 10 leads). **c** The anatomical sites in the brain of the leads E2 (red, insula epileptogenic cortex), E6 (yellow, peri-epileptogenic cortex), E7 (green, white matter), and E8 (blue, peri-epileptogenic cortex). **d** The HF activities recorded in leads E6, E7, and E8. **e** The energy changes on leads E6, E7, and E8. **f** The Sv DC on leads E2, E6, E7, and E8. The Sv DC variations in leads E6, E7, and E8 exhibit a consistent trend with a high level of correlation, while the Sv DC in lead E2 fluctuates around this trend, demonstrating a lower degree of fluctuation during the stable phase (light gray region) and a higher degree of fluctuation during the active phase (dark gray region). **g** The variations in energy (across the full frequency range, 0.01−300 Hz) between stable and active phases on different leads. The energies in the active phase are significantly higher than those in the stable phase (E2: *P* < 0.001; E6: *P* = 0.002; E7: *P* = 0.002; E8: *P* = 0.008). ^**^P < 0.01, ^***^P < 0.001. Sv DC slow-varying direct current

The high-frequency electrical activity recorded from an electrode contact located within the epileptogenic zone (E2) and a white matter contact outside the epileptogenic zone (E7) were compared (Fig. 2a–d). As shown, based on the characteristics of high-frequency activity, the epileptogenic zone (E2) exhibited 2 distinct phases: a stable phase and an active phase. During the stable phase, high-frequency activity was primarily confined to the epileptogenic zone, whereas in the active phase, it extended to the white matter outside the epileptogenic zone (Fig. 2a, d). During the interictal period, to better distinguish energy trends, we took the mean energy value of brain electrical activity from the hour preceding the observation period as the baseline. Periods where the energy increased by 100% compared with the baseline were defined as active periods. This allowed us to calculate the energy value changes for each electrode lead in each patient (Fig. 2a, e). To prevent interference from noise and other neural activities, we employed sliding window selection based on the mean and variance. Considering both time length and stability, we found that a 6-second time window provided a high level of discrimination. It was found that these energy changes are individualized and vary across different brain regions, time periods, and patients. We performed full-band energy calculations for the above contacts (Fig. 2a, e). The results show that the energy during the active phase is generally higher than during the stable phase (*P* < 0.01; Fig. 2g). The Sv DC variations recorded from the white matter contact outside the epileptogenic zone exhibit a consistent trend with a high level of correlation, whereas the Sv DC in the epileptogenic zone fluctuates around this trend, showing lower variability during the stable phase and increased fluctuation during the active phase (Fig. 2f). Furthermore, we found that this difference between the epileptogenic zone and the surrounding brain regions is a common phenomenon. The fluctuating patterns of Sv DC signals during the interictal period in 6 patients are illustrated in Additional file 1: Fig. S3. Consistent characteristics are visible: the Sv DC signals from leads outside the epileptogenic zone had similar trends and were consolidated, whereas the Sv DC signals from leads within the epileptogenic zone displayed more significant fluctuations (Fig. 2f). This pattern of Sv DC fluctuations observed within the epileptogenic zone suggests that Sv DC could serve as a potential indicator for localizing the epileptogenic zone.

### Volume conduction in seizure propagation

**Fig. 3:**
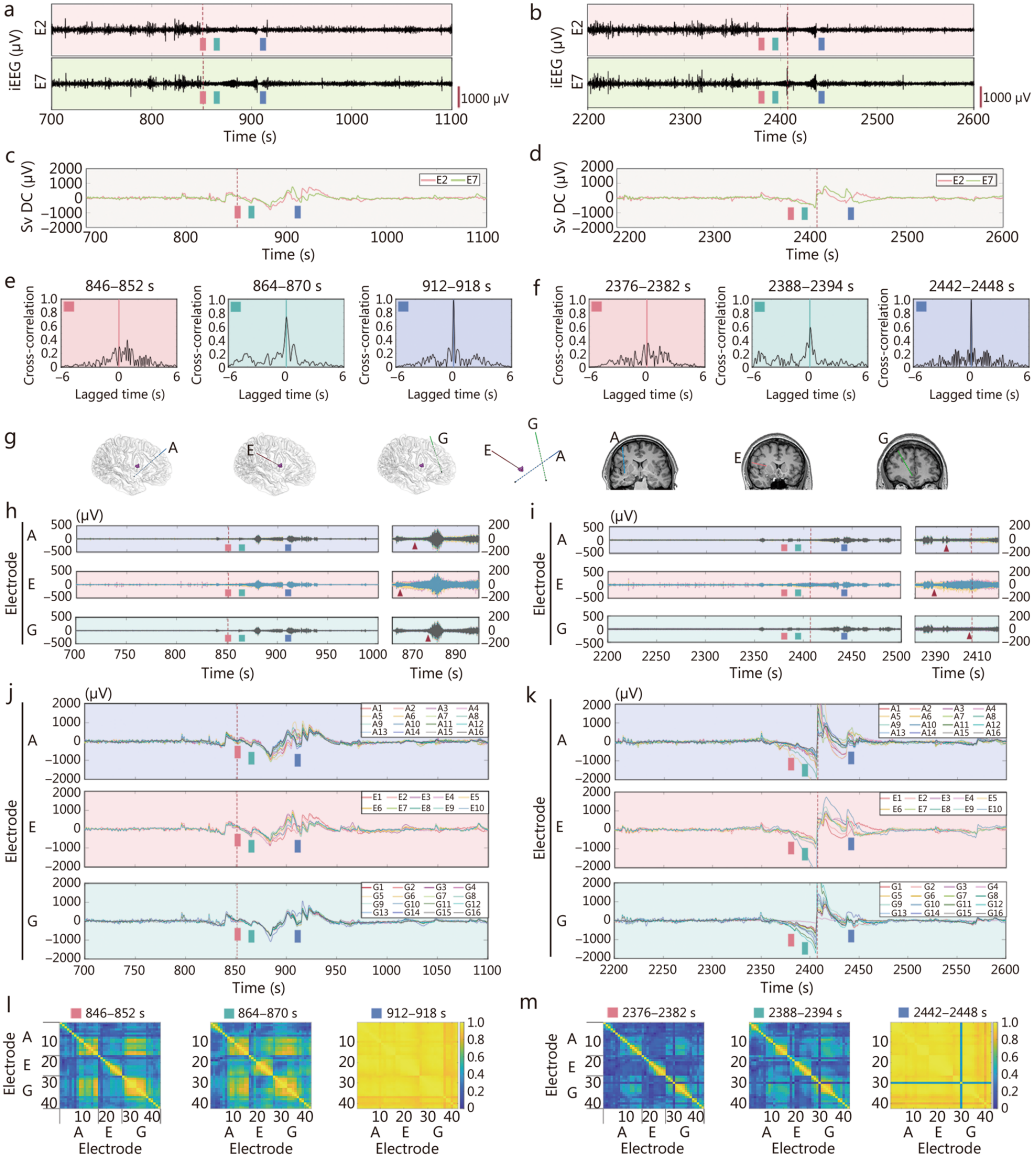
The conduction characteristics during seizures (taking patient B as an example). **a**, **b** The ictal activities (0.5−300 Hz) in 2 seizures on the leads E2 and E7. The amplitude scale is shown in the lower right corner. E2 lies in the epileptogenic cortex and E7 lies in white matter, the red dashed line indicating the seizure onset identified by clinical visual analysis. **c**, **d** The slow-varying direct current (Sv DC) during seizure in leads E2 (red) and E7 (green). **e**, **f** The cross-correlation between E2 and E7 during the seizure propagation. The 3 panels (red, green, and blue) represent the cross-correlation values in different stages during the 2 seizures propagation, respectively. At a certain time, the correlation between E2 and E7 lead is close to 1 (blue squares). **g** The position of the epileptogenic zone about the A, E, and G electrodes. The electrode A (16 leads) represents the electrode peri-epileptogenic zone. The electrode E (10 leads) represents the electrode entering epileptogenic zone, and electrode G (16 leads) represents the electrode distal to epileptogenic zone. **h**, **i** The high-frequency (HF) activities of the A, E, and G electrodes in the 2 seizures. Seizure onset low-voltage fast activity (LVFA, red triangles) appeared in the order of E, A, and G electrodes. **j**, **k** The characteristic of Sv DC in the A, E, and G electrodes. **l**, **m** Phase synchronization of the A, E, and G electrodes. The 3 panels represent the phase synchronization in the different stages during the 2 seizures’ propagation, respectively. At a certain time, the PLV values among the 3 electrodes are close to 1 (the blue line at lead 30 in Fig. 3 m is artifact). iEEG intracranial electroencephalography, Sv DC slow-varying direct current, PLV phase locking value

Taking 2 seizures from patient B as examples, it explains the pattern of volume conduction diffusion (Fig. 3). The full-band EEG activity and Sv DC signals recorded from within (E2) and outside (E7) the epileptogenic zone during these 2 seizures are shown in Fig. 3a–d, respectively. Following seizure onset, cross-correlation analysis revealed a progressive increase in the correlation between the epileptogenic cortex (E2) and the peripheral white matter (E7) of the E electrode, suggesting the diffusion of ictal activity along neural pathways during the seizure (Fig. 3e, f). The correlation subsequently increased significantly, indicating the emergence of volumetric conduction diffusion. Furthermore, a cross-correlation analysis was conducted across electrodes A, E, and G, which were located in different brain regions during the 2 seizures. The anterior contacts of electrode E were positioned within the epileptogenic zone, electrode A was located in the surrounding cortex, and electrode G was placed in a distant region from the epileptogenic zone (Fig. 3g). The high-frequency activity and Sv DC signals recorded from these 3 electrodes during the 2 seizures are shown in Fig. 3h–k, respectively. Network analysis utilizing electrode E (inside the epileptogenic zone), alongside electrode A (positioned near the epileptogenic focus) and electrode G (located at a distal site), revealed a progressive increase in correlation among the electrodes following seizure onset, ultimately achieving an extremely strong correlation indicative of complete synchronization (Fig. 3l, m). Overall, Fig. 3 illustrates an additional mechanism of seizure spread, volumetric conduction diffusion, alongside the conventional mode of propagation along neural pathways. In volumetric conduction diffusion, brain tissue functions as a volumetric conductor, resulting in a spatial distribution of the electric field throughout its volume. This pattern frequently occurs in the latter half of an episode.

### Cortical stimulation validates the Sv DC

**Fig. 4:**
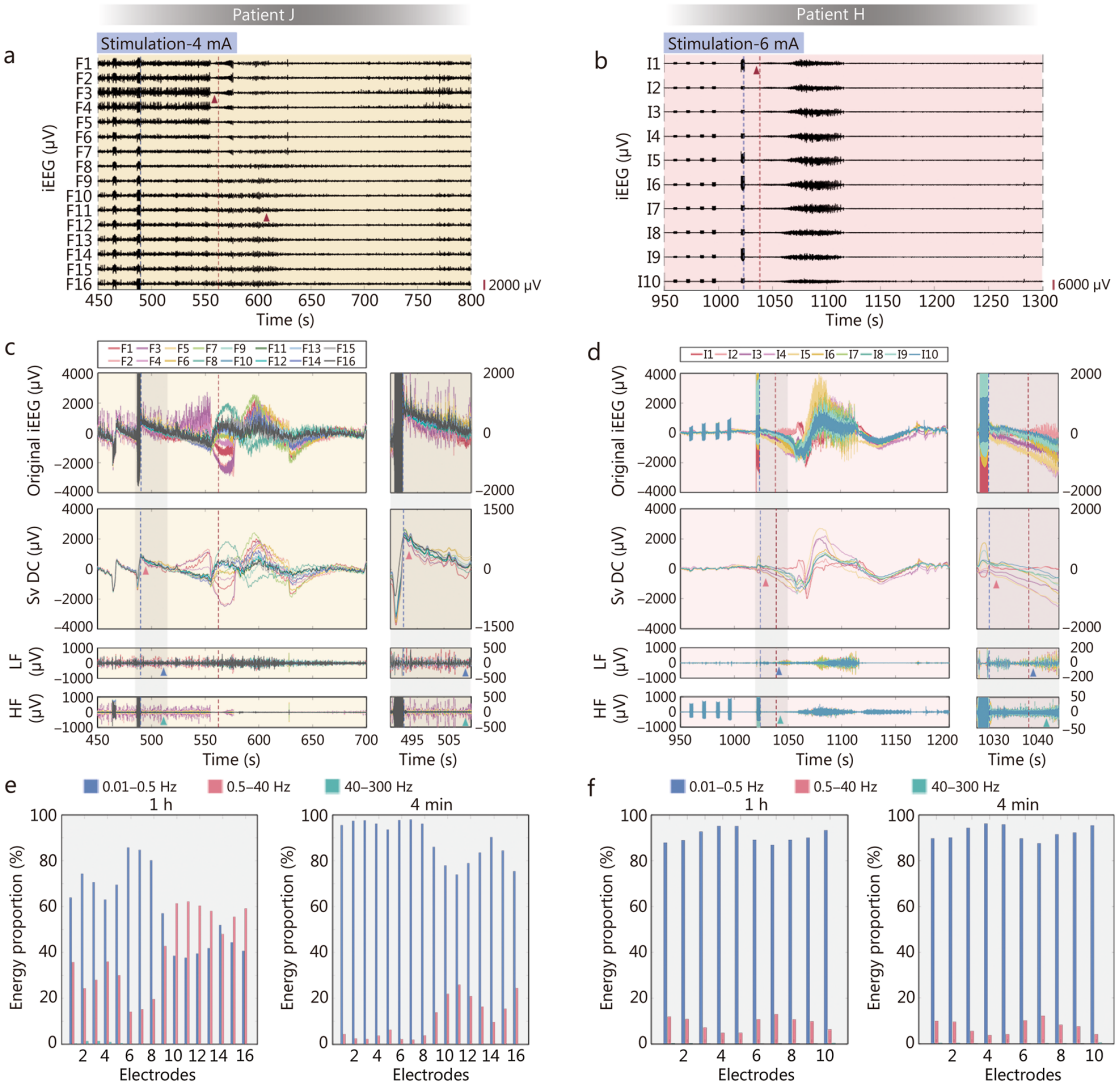
Characteristic of intracranial electroencephalography (iEEG) in seizures triggered by electrical cortical stimulation. The focal iEEG on electrodes F (16 contacts) and I (10 contacts) in patient J (**a**) and patient H (**b**) during the course in which electrical cortical stimulation induces seizures. The amplitude scale is shown in the lower right corner of panels. The blue dashed line indicates the point of time when the final electrical stimulation (4 mA, 6 mA) finished. The red dashed line shows the point in time at which the seizure began. In patient J, the low-voltage fast activity (LVFA) at seizure onset (red triangles) gradually spreads from the anterior leads to the posterior leads of the F electrode. On the contrary, the LVFA at seizure onset rapidly spread from the anterior leads to the rest of the leads in patients H. **c, d** The iEEG with different frequency bands during the 2 induced seizures. The full-band iEEG, slow-varying direct current (Sv DC), low-frequency (LF), and high-frequency (HF) were shown from top to bottom. The gray region on the right panel shows the Sv DC dispersed (red triangle) earlier than the onset change of LF (blue triangle) and HF (green triangle) in detail. **e, f** The energy proportion of a variety of frequency bands. The left panels show the energy proportion of each lead of electrode F in the 1 h and the 4 min’ EEG after the end of stimulation, respectively

During the preoperative assessment for mapping functional areas and epileptogenic zones via electrical cortical stimulation, stimulating the epileptogenic cortex can induce auras or seizures. We analysed the EEG data of seizures induced by electrical cortical stimulation via the aforementioned EEG processing methods (Fig. 4a, b). The results showed that after the termination of electrical stimulation in the epileptogenic zone, a distinct Sv DC shift and dispersion immediately appeared at or near the stimulation site, followed by continuous expansion of this dispersion until the onset of a seizure (Fig. 4c, d). These findings strongly validate our theory that Sv DC shift and dispersion are characteristics of seizure onset. As shown in Fig. 4c and d, during the mapping process with gradually increasing current intensity, seizures occurred only when the Sv DC shift and dispersion were clearly observed.

In patient J (Fig. 4a), after the seizure was triggered by cortical electrical stimulation, the seizure activity gradually spread to surrounding areas, whereas in patient H (Fig. 4b), the seizure activity rapidly involved adjacent tissues, leading to volume conduction. These 2 patterns are similar to the natural seizure characteristics of patients W and B (Fig. 1). Full-band energy analysis revealed that the energy proportion during the 4 min seizure period was significantly greater than the energy proportion during the 1 h period, including the seizure period, which is consistent with the patterns observed in natural epileptic seizures (Fig. 4e, f).

## Discussion

In recent years, intracranial electrode recording techniques, particularly SEEG electrode recording, have played a significant role in the diagnosis and treatment of epilepsy, providing high temporal and spatial resolution data on brain electrical activity. However, the correlation between the dynamic topological structure of brain electrical spread and fundamental biophysical mechanisms remains an area of active investigation, with some aspects still requiring further clarification [12]. Brain electrical signals, as complex and time-varying alternating current signals, exhibit spontaneity [21], causality [22], and event responsiveness [23] while also containing slow, nonperiodic DC components. Owing to the inherent weakness of brain electrical signals and their susceptibility to external interferences, particularly those affecting DC activity, such as electrode polarization, baseline shifts in biopotentials, and DC shifts, these interference factors can introduce errors into the analysis of brain electrical signals. Therefore, filtering out DC components has become a routine preprocessing step in brain electrical signal processing. The purpose is to improve signal quality, enhance the accuracy of spectral analysis, reduce the baseline shift, simplify processing procedures, and ensure that the signal meets the zero-mean assumption. However, in this study, we revealed a unique biophysical phenomenon observed during seizures: the Sv DC field. This phenomenon exhibits a dynamic behavior pattern of dispersion to convergence, combining the stability of physical DC electric fields with the slow variability of biological processes, which provides a new perspective for exploring the biophysical mechanisms of seizures.

Compared with the single-lead icDC phenomenon documented in the previous literature [16], the Sv DC tends to exhibit more holistic shifts rather than divergence in the face of interference. Its divergent characteristics are a unique phenomenon resulting from local potential differences, objectively reflecting the slow-varying characteristics of spatial electric fields over time. Therefore, the Sv DC not only provides a new entry point for the biophysical study of seizures but also has the potential to serve as a more specific and stable biomarker, facilitating a deeper understanding and more effective diagnosis of seizures. By analyzing long-term intracranial recordings, including both interictal and ictal iEEG data from 24 patients with epilepsy, we confirmed that the Sv DC component holds an important position in terms of energy in both contexts, particularly dominating during seizures. According to system theory, this dominant component has a strong influence on other components. However, in clinical EEG analysis, the Sv DC is usually directly filtered out, which is undoubtedly a significant loss in brain electrical analysis. Therefore, future research should give more attention to the biophysical significance of Sv DC in seizures and its potential clinical application value.

Through detailed decomposition of brain electrical activity, the study revealed that simultaneous analysis of the Sv DC components, low-frequency components, and high-frequency components at the same time points can provide more comprehensive and accurate EEG informations. The identified onset point of Sv DC component dispersion can serve as an early indicator of the development of an epileptic seizure. Furthermore, the study indicates that epileptic seizures often originate during periods of energetic activity. Therefore, monitoring energy and Sv DC fluctuations during these active periods provides a means for seizure monitoring to make epileptic seizures predictable.

The study revealed that the Sv DC component remains stable most of the time but fluctuates during specific periods, particularly in epileptogenic zones. It is believed that the dispersion of the Sv DC is closely associated with changes in potential differences. Therefore, we prefer to describe this component as an Sv DC field to distinguish it from a pure DC. Sv DC dispersion occurs only when there is a significant potential difference, and the degree of dispersion correlates with the magnitude and duration of the potential difference change. Brief changes in potential differences result in transient fluctuations in the Sv DC, whereas intense and sustained changes may trigger epileptic seizures. Moreover, leads located in the epileptogenic zone are more prone to Sv DC dynamic fluctuations accompanied by energy changes, whereas Sv DC fluctuations in other brain regions far from the epileptogenic zone are smaller. These findings suggest that leads with significant Sv DC fluctuations can serve as potential markers to help locate the epileptogenic zone. If the epileptogenic zone can be located through such markers during the interictal period, it will significantly reduce the dependence on ictal recordings, optimize the surgical evaluation process, and decrease patient risk and suffering. Therefore, real-time monitoring of the Sv DC dispersion can also be used as an important indicator for seizure monitoring and localization of seizure onset. This also offers a novel approach for closed-loop neuromodulation.

The spatial dynamic changes in the Sv DC component provide a window for observing the evolution of local electric fields, enabling us to establish a mapping relationship between microscopic ion electric fields and induced currents. These findings indicate that changes in potential differences may contribute to the cause of Sv DC activity dispersion and could play a significant role. We hypothesize that these fluctuations in the Sv DC component result from pathological abnormalities that lead to localized ion transport disruptions, subsequently generating abnormal electric field phenomena akin to a battery effect. When the potential difference is sufficiently strong or there are specific pathological changes in the surrounding tissue, the local electric field may exhibit volume diffusion, similar to a battery effect. This diffusion allows large-scale brain tissue to act as a volume conductor, characterized by highly correlated oscillatory diffusion in the cortex and white matter of relevant brain regions. This view is also supported by the cortical electrical stimulation conducted in this study, where applying a certain current intensity to the local cortex induces local potential differences. If these potential differences result in significant electric field coupling and diffusion, a clinical seizure can occur. Stronger currents applied through electrical cortical stimulation generate greater potential differences, leading to a larger induced electric field, which is more likely to trigger seizures and lead to volume conduction in brain tissue. This finding effectively validates our perception that the local potential difference is the cause of seizure occurrence in epilepsy.

Recent in vivo and in vitro electrophysiological experimental results support our view that electric field coupling is a key mechanism of nonsynaptic neural propagation, enabling the volume conduction of electric fields within the brain, which is also an important pathway for the spread of epileptic activity in the brain [24–29]. Furthermore, we recognize that the causes of brain tissue volume conduction may be multifaceted. In addition to electric field intensity, factors such as the pathological characteristics of local lesions and abnormalities in local network connections might also significantly influence this phenomenon. These findings provide a new perspective for a deeper understanding of the biophysical mechanisms underlying epileptic seizures.

**Fig. 5:**
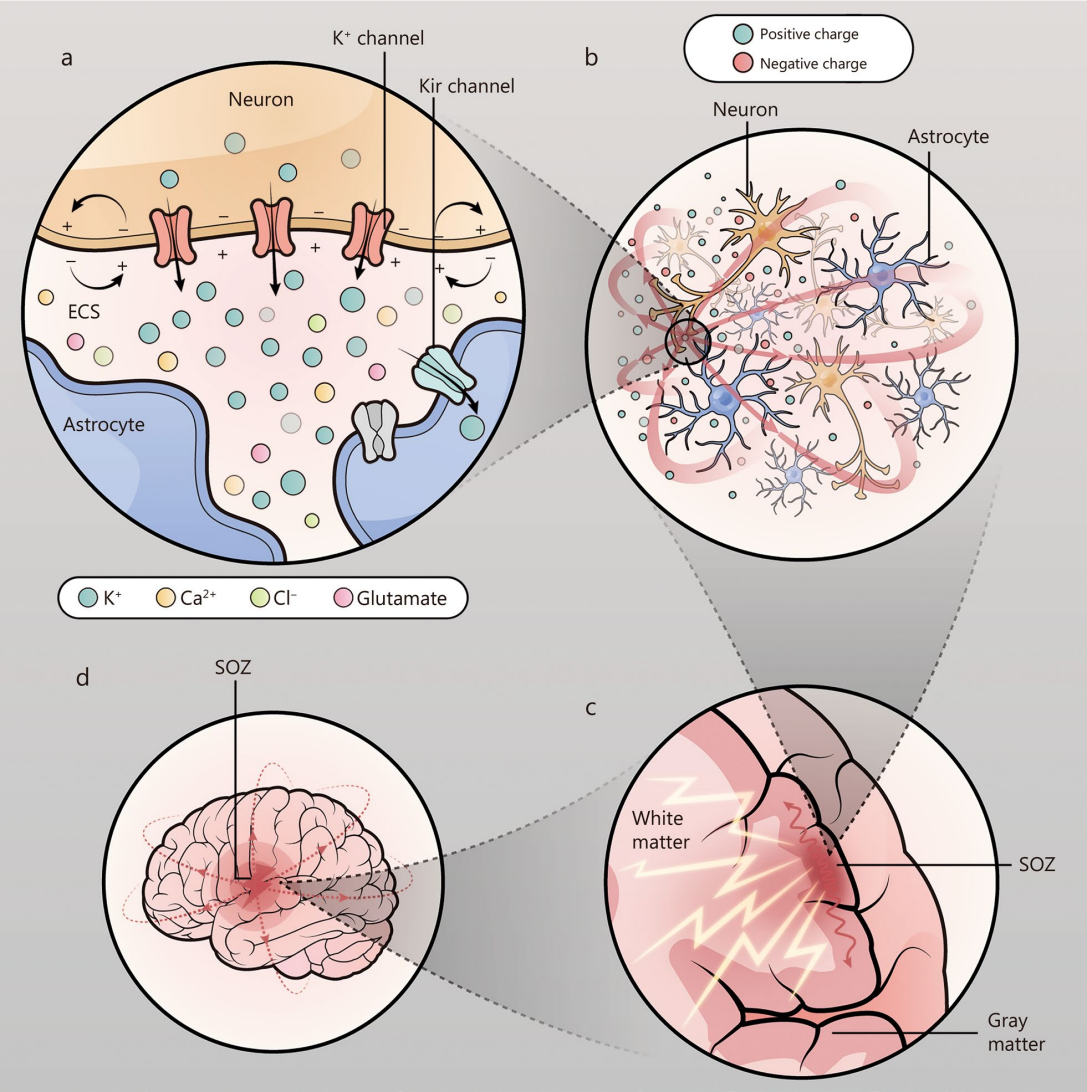
Schematic of local potential difference inducing seizures. **a** A significant quantity of neurons produced action potentials simultaneously which cause the flow of K^+^ into the extracellular space (ECS) through ion channels. Pathological factors, including abnormalities in the extracellular space or dysfunction of the Kir channels (gray) in cell membranes of adjacent astrocytes, prevent the majority of K^+^ in the ECS from entering the astrocytes promptly, resulting in its accumulation in the ECS. **b** A large amount of K^+^ accumulates in the ECS, causing a change in the local extracellular environment and generating an electrical potential difference relative to the surrounding tissues, which causes the electric field to propagate outward (red line). **c** The electric field generated within the cortical epileptogenic focus diffuses in an outward direction through electric field coupling. Under certain conditions, the phenomenon of volumetric diffusion may develop (lightning icon). **d** Diffusion of the electric field (red dashed line: spreading of the electric field) from the epileptogenic zone to peripheral or extensive brain areas. SOZ seizure onset zone

The causes of the local potential differences that generate the Sv DC have yet to be elucidated. A study has shown that when a group of neurons produce action potentials in a short period, a rapid increase in extracellular K^+^ develops. Simultaneously, this excitatory activity triggers the massive activation of interneurons, resulting in synaptic inhibition, which further increases the extracellular K^+^ concentration [30, 31]. Extracellular K^+^ accumulates in the extracellular space (ECS), which is the narrow gap between neurons and astrocytes [32]. However, pathogenic factors, such as injury or developmental abnormalities, can affect the ECS within the epileptogenic zone and cause astrocytes to become dysfunctional. Studies have confirmed the abnormal expression and loss of function of Kir4.1 in various animal models of acquired epilepsy and in tissues from epileptic patients [33–35]. Therefore, a large amount of K^+^ accumulates in the ECS within the epileptogenic zone in a short time without effective buffering, this compounded by changes in ions and metabolites such as Cl^−^, Ca^2+^, glutamate, and others [36–38], leading to the collapse of the extracellular environment, significant changes in local field potentials within a short period, and thus notable potential differences. In several studies on epilepsy animal models, obvious DC electrical activity was also observed in the early stages of seizures [16, 39]. Therefore, the initial potential differences in seizures reflect the comprehensive result of interactions involving multiple neuron-glia factors [16, 31, 40, 41]. When this potential difference generates an electric field with surrounding cellular groups [26, 29], this electric field passively depolarizes the surrounding neurons, repeating in a cycle and spreading to the surroundings through electric field coupling. Combining the findings of our study with previous research, we provide a preliminary explanation for this inference in Fig. 5.

This study proposes the concept of potential differences during epileptic seizures and their potential value on the basis of the Sv DC shift and dispersion behavior derived from the iEEG signals of epileptic patients. However, further validation is needed, which constitutes a limitation of this study. For example, previous studies with microelectrodes showed that increased extracellular K^+^ levels can trigger epileptiform discharges [30, 31, 42]. In contrast, our study with macro-electrodes demonstrated large-scale Sv DC fields. Whether changes in ion concentration can generate such fields remains unclear, as this was not the focus of our study. Furthermore, current electrode technology limits the simultaneous in vivo measurement of microscopic ion dynamics and macroscopic field potentials. Future advances, such as dual-mode electrodes, may enable integrated micro- and macro-electrode recordings that provide deeper insight into the quantitative relationship between ion dynamics and large-scale fields. We propose that frequent Sv DC fluctuations and energy changes during the active phase serve as key indicators for epilepsy monitoring, seizure prediction, and epileptogenic zone localization. Further validation is needed. Our next steps involve studying the link between extracellular ion concentration, local field potentials, and Sv DC fields in animal models.

## Conclusions

The study described a characteristic biophysical phenomenon in epileptic seizures: the Sv DC field. The Sv DC dominates the energy changes during epileptic seizures, combining the stability of a physical DC with the slow nature of biological changes, reflecting the dynamic process of relative changes in bioelectric fields, and exhibiting shifting-dispersing variations on different leads during epileptic seizures. This dynamic behavior of the Sv DC is speculated to be attributed to changes in local potential differences. The discovery of the Sv DC field provides a new perspective for understanding the biophysical mechanisms of epileptic seizures.

## Supplementary Information

**Additional file 1.** Clinical data collection. **Fig. S1** The phenomenon of Sv DC in 24 patients. **Fig. S2** The energy proportion of different frequency EEG. **Fig. S3** The Sv DC within or outside the epileptogenic cortex.

**Additional file 2.** **Table S1** The energy data of different frequency EEG activities (1 h data, including 4 min episodes). **Table S2** The energy data of different frequency EEG activities (4 min episodes).

## Data Availability

The data that support the findings of this study are available from the corresponding author upon reasonable request.

## Abbreviations

CT: Computed tomography
DC: Direct current
ECS: Extracellular space
EEG: Electroencephalography
HFOs: High-frequency oscillations
icDCs: Ictal direct current shifts
iEEG: Intracranial electroencephalography
LVFA: Low-voltage fast activity
MRI: Magnetic resonance imaging
PLV: Phase locking value
SEEG: Stereo-electroencephalography
Sv DC: Slow-varying direct current
