# Supplementary material for: Epileptic seizure biophysics: the role of local voltage difference
Source: Mil Med Res. 2025 Jul 11;12:35. doi: 10.1186/s40779-025-00620-4 (PMC12247268; doi:10.1186/s40779-025-00620-4)
Supplement: Supplementary file 1 — Additional file 1. Clinical data collection. Fig. S1 The phenomenon of Sv DC in 24 patients. Fig. S2 The energy proportion of different frequency EEG. Fig. S3 The Sv DC within or outside the epileptogenic cortex. [file 40779_2025_620_MOESM1_ESM.pdf]

Clinical data collection

Presurgical evaluation

Each patient underwent interictal/ictal scalp electroencephalography (EEG) recordings via a video-EEG monitoring system (Neuracle, China), with electrodes placed according to the international 10 − 20 system. The duration of scalp video EEG monitoring ranged from 3 to 14 d, and at least 2 habitual seizures were recorded for each patient. All the information acquired via interictal and ictal EEG, along with the seizure semiology of each patient, were analyzed to assist in locating the epileptogenic zones (the primary organization of the epileptogenic activity) [1].

All patients underwent a high-resolution magnetic resonance imaging (MRI) protocol using a 3.0-T MR scanner (Siemens, Germany or United Imaging, China). This protocol included conventional spin-echo T1-weighted axial, sagittal, coronal, and T2-weighted axial sequences (section thickness of 5 mm, image gaps of 1 mm). Additionally, fluid-attenuated inversion recovery images were obtained at a thickness of 1 mm. Furthermore, three-dimensional anatomical T1-weighted axial, sagittal, and coronal sequences were acquired, covering the whole-brain volume with a 0.8-mm section thickness.

Although MRI plays a significant role in the diagnosis of epileptic foci, it may yield either ambiguous structural abnormalities or completely negative results for some patients with focal epilepsy [2-4]. To address this limitation, positron emission tomography was performed on all patients to facilitate the localization of the epileptogenic zones.

The localization of epileptogenic zones, as well as the relationship between the epileptogenic zones and the functional cortex, was fully evaluated by a specialist group (including at least 2 neurologists, 1 neurophysiologist, and 1 neurosurgeon) when the clinical data were collected. As suggested by the specialist group, these patients were recommended for intracranial recording with

stereo-electroencephalography (SEEG) electrodes because insufficient information was obtained from non-invasive examination.

The procedure of SEEG

A team of experienced neurologists and neurosurgeons formulated a hypothesis regarding the localization of the epileptogenic zone and designed a SEEG implantation plan for each patient based on the results of non-invasive presurgical evaluations. The plan included the number of SEEG electrodes, their anatomical targets, trajectories, and angles. Subsequently, the SEEG electrodes, consisting of intracerebral multiple-contact electrodes (10 − 16 contacts; length: 2 mm; diameter: 0.8 mm; spacing: 1.5 mm), were implanted stereotactically using a robotic arm-assisted system (Sinovation Medical Technology, China). Postsurgical computed tomography (CT) scans were performed to verify the locations of the contacts and to rule out new intracranial hemorrhage.

The reconstruction and localization of the deep electrodes were performed in accordance with established protocols [5]. In brief, on the basis of MRI before implantation, we used the FreeSurfer image analysis suite (https://surfer.nmr.mgh.harvard.edu/) to reconstruct the cortical surface. The postoperative CT image was then coregistered to the T1 image and converted into Talairach space. Electrodes were detected by clustering-based segmentation. The direction, starting point, and deepest point of the electrodes were verified via presurgical designation. The trajectory of each electrode was fitted to a curve, and the contacts were represented by equally spaced dots.

Long-term intracranial EEG recording

Intracranial electroencephalography (iEEG) monitoring was performed via a 128- or 256-channel amplifiers (selected based on the total number of SEEG electrode contacts for each patient). iEEG data

were recorded using the Neuracle recording system and sampled at 1024 Hz. The duration of iEEG monitoring ranged from 5 to 16 d, and at least 2 habitual seizures were recorded for each patient. SEEG data were processed with a typical bipolar montage. The onset of each seizure was visually assessed and marked on the ictal EEG, on the basis of the changes in the rhythm of electrical activity, by 2 experienced neurophysiologists. These marked EEG data were provided to a specialist preoperative evaluation team to localize the epileptogenic zones comprehensively. These iEEG data, including those from the interictal and ictal periods, are saved and backed up for further analysis and research.

Electrical cortical stimulation

Patients underwent electrical cortical stimulation during long-term iEEG monitoring for localizing the eloquent cortex or identifying the epileptogenic zones. Stimulation was applied using a biphasic wave with the following parameters: pulse width of 0.2 ms, frequency of 50 Hz, and duration of 3 s. Referring to our clinical protocol and previous publications [6], we aimed to minimize the pathological after-discharges, which could impact the accurate identification of the epileptogenic zone, and reduce the induction of epileptic seizures. Our current intensity commenced at 0.5 mA and increased by 0.1 or 0.2 mA, up to a maximum of 6 mA [7]. For each electrode contact, direct electrical cortical stimulation was terminated when a definite aura or seizures were elicited or when the current intensity reached 6 mA.

References

1. Gonzalez-Martinez JA. The stereo-electroencephalography: the epileptogenic zone. J Clin Neurophysiol. 2016;33(6):522-29.
2. Mauguière F, Ryvlin P. The role of pet in presurgical assessment of partial epilepsies. Epileptic Disord. 2004;6(3):193-215.
3. Rheims S, Jung J, Ryvlin P. Combination of PET and magnetoencephalography in the presurgical assessment of MRI-negative epilepsy. Front Neurol. 2013;4:188.
4. Willmann O, Wennberg R, May T, Woermann FG, Pohlmann-Eden B. The contribution of ^{18}F-FDG PET in preoperative epilepsy surgery evaluation for patients with temporal lobe epilepsy a meta-analysis. Seizure. 2007;16(6):509-20.
5. Yu T, Wang X, Li Y, Zhang G, Worrell G, Chauvel P, et al. High-frequency stimulation of anterior nucleus of thalamus desynchronizes epileptic network in humans. Brain. 2018;141(9):2631-43.
6. Fox KCR, Yih J, Raccah O, Pendekanti SL, Limbach LE, Maydan DD, et al. Changes in subjective experience elicited by direct stimulation of the human orbitofrontal cortex. Neurology. 2018;91(16):e1519-e27.
7. Prime D, Rowlands D, O'Keefe S, Dionisio S. Considerations in performing and analyzing the responses of cortico-cortical evoked potentials in stereo-EEG. Epilepsia. 2018;59(1):16-26.

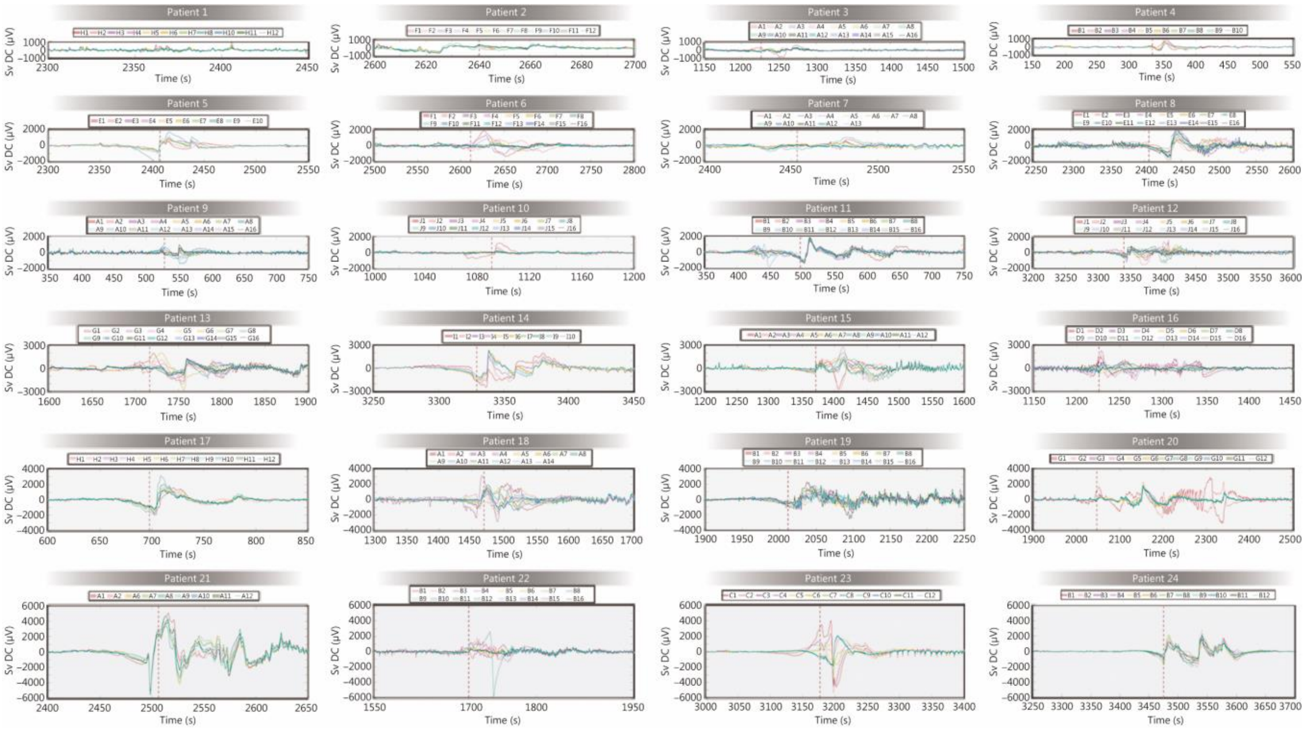

Fig. S1 The phenomenon of slow-varying direct current (Sv DC) in 24 patients. The shift and dispersion of the Sv DC between different leads from the seizure onset electrodes in 24 patients. One seizure from each patient was selected and shown in this Figure. Several leads were removed due to artifacts in 4 patients (Lead 9 in patient 1; Lead 14, 15 and 16 in patient 7; Lead 15 and 16 in patient 18; Lead 3, 4 and 5 in patient 21)

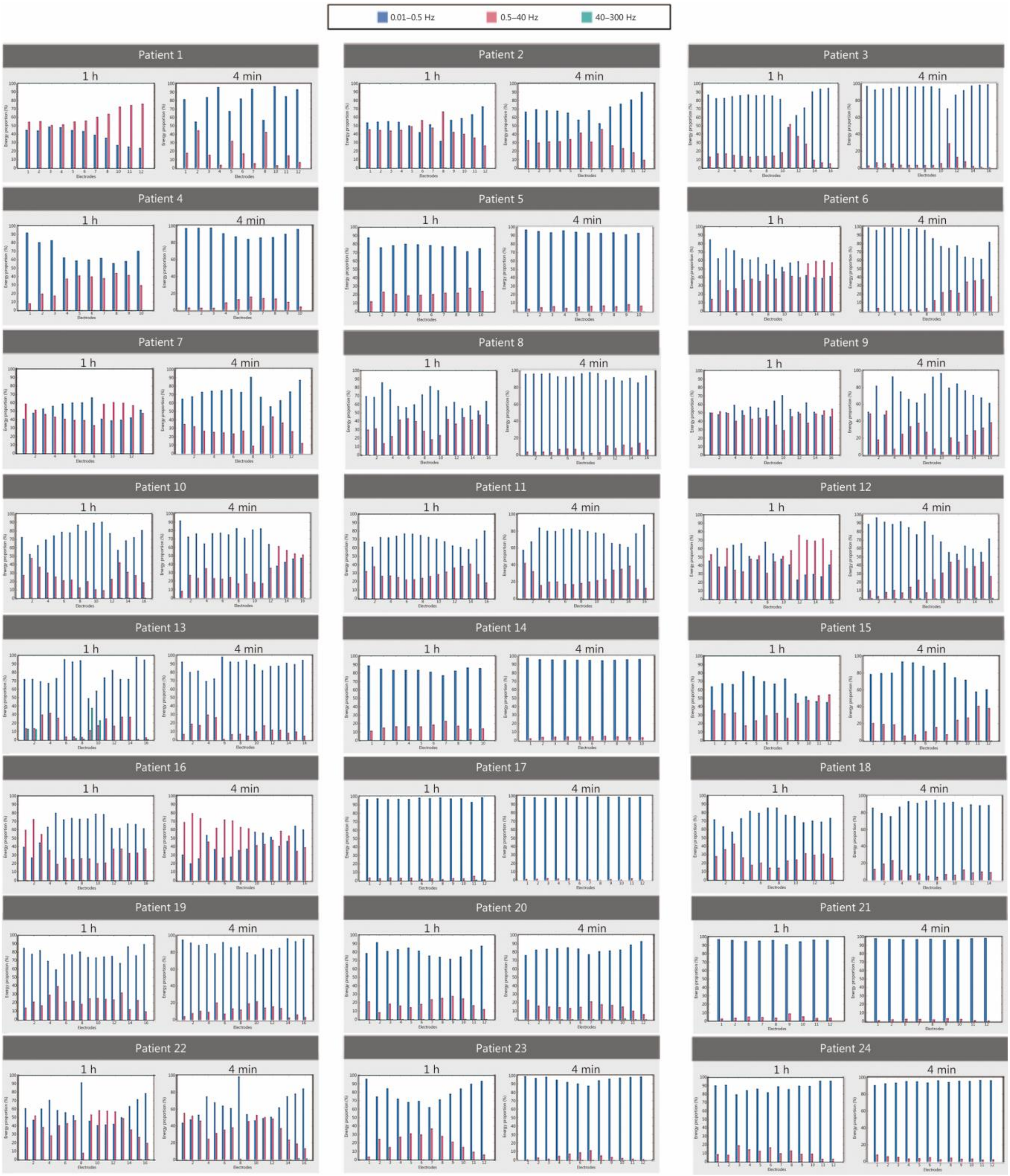

Fig. S2 The energy proportion of different frequency electroencephalography (EEG). The energy proportion of different frequency EEG on the seizure onset electrode in 24 patients. One seizure from each patient was selected and shown in this Figure. The left panels display the energy proportion of each electrode lead for 1 h EEG data (including the ictal period), whereas the right panels show the energy proportion of each electrode lead for 4 min EEG during the ictal period (2 min before and 2 min after the onset). The blue color denotes the slow-varying direct

current (Sv DC) component (0.01 − 0.5 Hz), the red color signifies the 0.5 − 40 Hz component, and the green color indicates the 40 − 300 Hz component. Several leads were removed due to artifacts in 4 patients (Lead 9 in patient 1; Lead 14, 15 and 16 in patient 7; Lead 15 and 16 in patient 18; Lead 3, 4 and 5 in patient 21)

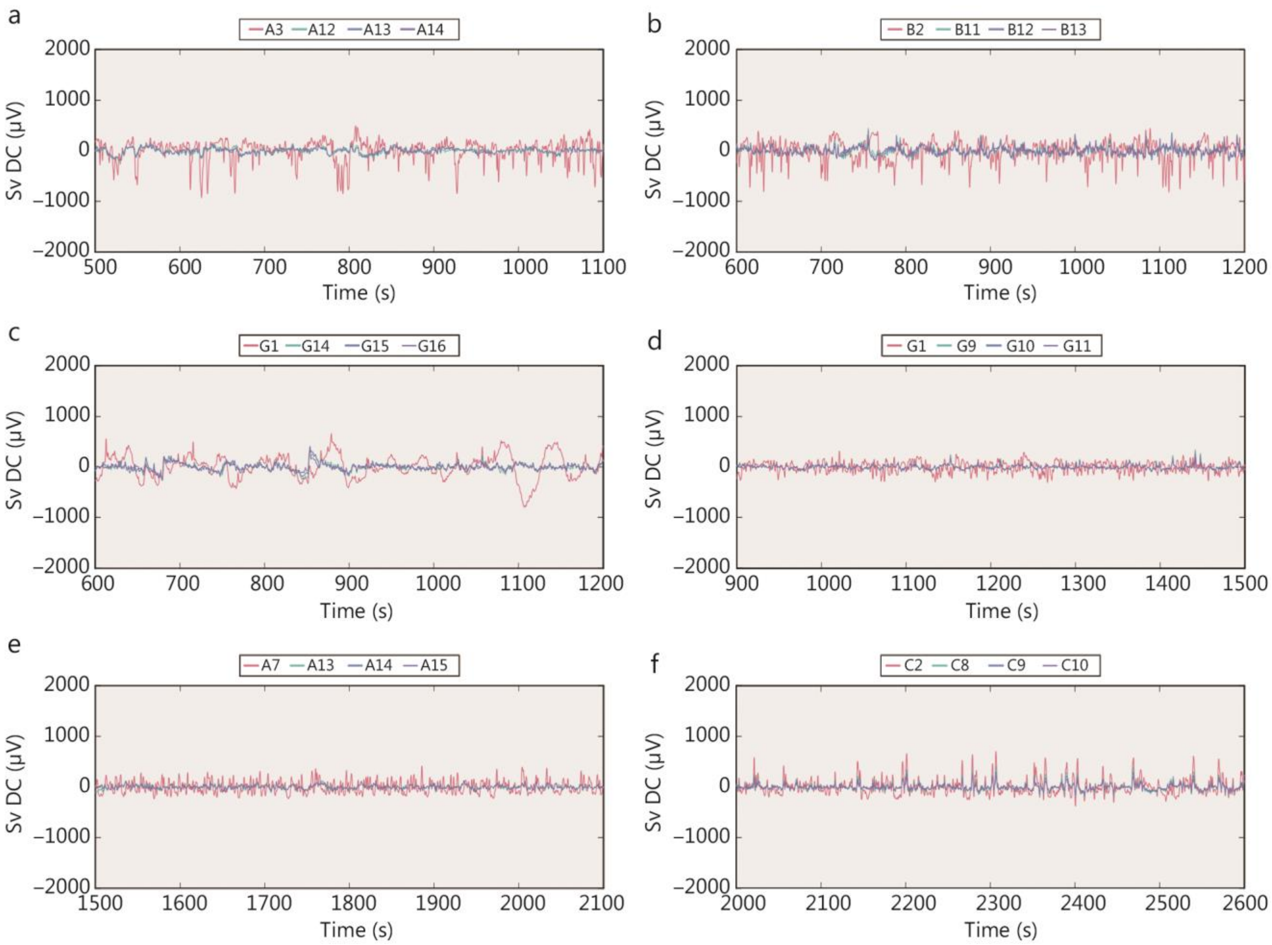

Fig. S3 The slow-varying direct current (Sv DC) within or outside the epileptogenic cortex. The Sv DC changes of the leads within the epileptogenic cortex (red) compared to the leads surrounding or distal the epileptogenic zone (green, blue, and purple). The Sv DC on the leads outside the epileptogenic zone exhibits a consistent pattern with significant aggregation, whereas the Sv DC on the lead within the epileptogenic cortex fluctuates around this pattern. This typical Sv DC fluctuating phenomenon was selected and shown in 6 patients
